# Challenges in Understanding Numerical Learning: Editorial for *Brain Sciences* Special Issue “Neurocognitive Signatures of Math (Learning) across the Lifespan and Their Interrelation with Other Aspects of Cognition and Emotion”

**DOI:** 10.3390/brainsci13030420

**Published:** 2023-02-28

**Authors:** Elise Klein, Laura Zamarian, Liane Kaufmann

**Affiliations:** 1 CNRS, LaPsyDÉ, Université Paris Cité, La Sorbonne, 75005 Paris, France; 2 Leibniz-Institut fuer Wissensmedien, 72070 Tuebingen, Germany; 3 Department of Neurology, Medical University of Innsbruck, 6020 Innsbruck, Austria; 4 Department of Psychology, University of Innsbruck, 6020 Innsbruck, Austria

## 1. Introduction

Living in our information- and technology-driven society at the beginning of the 21st century requires the ability to understand and handle numbers not only for a successful career but also for coping with everyday life tasks. Deficient numerical skills can be the result of difficulties in acquiring math competencies (as in developmental dyscalculia, DD) but can also stem from acquired brain damage. In either case (and regardless of the neurocognitive signatures that seem to be distinguishable in developmental and acquired dyscalculia [1]), poor numerical skills negatively impact the affected individual and are associated with higher socioeconomic costs (due to low academic and occupational performance, the need for specific numerical skill interventions, and often related emotional problems such as math anxiety; for a review on societal and educational implications of low numeracy, see, for instance, [2]; and for a novel view on a cerebellum-driven social basis of math learning and its implications for education, see [3]).

In recent years, the neurocognitive foundations, predictors, and compensatory (reorganizational) mechanisms of number processing and math learning have gained increasing research interest at both personal and societal levels. Following this trend, we invited empirical and theoretical contributions for a Special Issue on Neurocognitive Signatures of Math (Learning) Across the Lifespan and Their Interrelation with Other Aspects of Cognition and Emotion. We are grateful to all authors for their high-quality contributions, the reviewers for their constructive comments and suggestions in the interactive peer-review process, and the publisher’s editorial team for the support. 

The different contributions nicely illustrate that the construct *Math Learning* does not denote a unitary, clearly circumscribed, and comprehensive entity. Instead, the experimental and review articles clearly suggest that it is important to consider different empirical and theoretical perspectives evaluating domain-general (e.g., executive function, emotional processing, or spatial abilities) but also domain-specific (e.g., basic numerical abilities) determinants of typical and atypical number processing across the lifespan.

## 2. Typical Numerical Development

### 2.1. Domain-General and Domain-Specific Influences

A first set of studies investigated domain-general as well as domain-specific influences on *typical numerical development*. In a first study with 3-year-old preschoolers, Cuder et al. [4] successfully employed a numerical training video to improve counting skills, intervention effects being visible even six months after the intervention. Moreover, the numerical training had transfer effects to cardinality understanding, while knowledge of the number line was unaffected. Poltz and colleagues [5] investigated the developmental trajectories of spontaneous focusing on numerosity (SFON) in preschool to second-grade school children. While SFON was found to facilitate numerical concepts in pre-school-age children, it had—contrary to previous findings—only indirect effects on children’s arithmetical development in the first and second grade. On the neuro-functional level, Van Bueren and colleagues [6] examined the underlying neurophysiological effects of the mathematical ability in children and adults using electro-encephalography (EEG). While the authors did not find a mediation of working memory and number sense in children or adults when using a traditional analysis approach, they were able to show that aperiodic activity rather than periodic activity seemed to be linked to mathematical ability when using a parameterization method. Finally, in a study on primary school children from grade 1 to 6, Gliksman and colleagues [7] demonstrated that the BGU-MF, a math fluency test initially developed and evaluated for adults, is a valid tool not only for adults but also for primary school children. 

### 2.2. Influences of Embodied Cognition 

A second set of studies investigated the neuro-cognitive influence of embodied cognition on *typical numerical development*. Employing EEG in 3-to-4-month-old infants, Decarli et al. [8] found evidence for a neuro-functional link between numerosity processing and hand action processing. With the help of functional near-infrared spectroscopy (fNIRS) in a mental arithmetic training in first graders, Artemenko et al. [9] were able to link the effects of a finger-based training on mental arithmetic (i.e., training-induced facilitation of subbase-5 carry operations) and the activation in the sensorimotor cortex in trained children, suggesting training-induced sensorimotor plasticity in brain development. Thus, the results of both studies support an embodied perspective on the representation of numbers already in early infancy.

## 3. Typical Numerical Processing in Children and Adults

### 3.1. Magnitude Estimation

Another set of studies focused on magnitude estimation in *typical numerical processing*. On the behavioral level, Feldman and Berger [10] showed in first- to third-grade children that performance in the number line estimation task is best described by a sigmoidal function with flexible breakpoints that incorporates features of both the two-linear and the proportion judgment models. This function showed two developmental leaps: a first leap when children learned to divide the number line into two segments; and a second leap when anchor points were used (left endpoint in first grade, right endpoint in second grade, midpoint in third grade). Interestingly, the use of anchor points was also associated with faster responses in mental arithmetic. On the neural level, Ashkenazi et al. [11] evaluated the neural networks underlying two different computation estimation strategies using functional magnetic resonance imaging (fMRI) in adults: approximate calculation and sense of magnitude intuitive representation. Approximate calculation was associated with stronger magnitude-specific activation in multiple parietal regions than sense of magnitude intuitive representation, while the inferior frontal gyrus seemed to be essential for both strategies. Finally, in a second fMRI study in adults, Ashkenazi et al. [12] demonstrated that the neurocognitive correlates of continuous magnitude estimation (mostly time) and discrete numerical estimation are distinguishable regarding frontal activation patterns. 

### 3.2. Emotional Influences on Math Learning

Another set of behavioral studies extends the focus on *emotional influences such as math anxiety on math learning.* In a first study, Maldonado et al. [13] were able to show that higher math anxiety in adults was associated with lower math scores and less precise numerosity estimation, but not with lower visuo–spatial working memory. Furthermore, math anxiety proved to be a mediating factor between numerosity estimation precision and math abilities. Additionally, in adults, Daches Cohen and colleagues [14] found that state (but not trait) math anxiety was linked to anxiety predisposition, the subjective valence of math information, and difficulties in emotional regulation. Thus, the authors proposed that anxiety predisposition and emotion regulation are important determinants of math anxiety. Finally, examining the interaction between anxiety and sustained attention for math learning and performance in children (fourth and fifth graders), Orbach and Fritz [15] observed a negative correlation between state anxiety prior to the math test and arithmetic achievements. Sustained attention, in particular, proved to be a strong predictor of arithmetic achievement and a moderator of the anxiety–performance interaction. 

## 4. Atypical Numerical Processing in Children and Adults

### 4.1. Developmental Dyscalculia

A set of four studies investigated *atypical numerical processing* with a particular focus on developmental dyscalculia (DD). Using a numerical cognition battery for children, Santos and colleagues [16] estimated the prevalence of DD between 4.6% (when the fifth percentile was taken as criterion) and 7.4% (when the z-score was applied) in children aged 7–12 years in a country (Brazil) with generally low educational attainment. Based on their findings and theoretical considerations, the authors suggest applying the first criterion (i.e., the fifth percentile) whenever the context is an educational one and the second criterion (i.e., the z-score) in a clinical context. In the study by Vigna et al. [17] the impact of DD on everyday life in young adults was evaluated behaviorally. The authors found poorer arithmetical skills (such as time and measure estimation, money usage in real-world numerical tasks, preserved distance estimation) in both formal and informal everyday settings. Particularly, DD was related to emotional problems and negatively impacted academic and occupational decisions. Goebel et al. [18], on the other hand, turned to the neural level in their fMRI single case study. In a case of an adult with DD they investigated fact retrieval (multiplication) and calculation (subtraction) and found two dissociable neurofunctional networks, which, however, were only processing-specific but not operation-specific. Depending on proficiency, for instance, procedure-based strategies and their associated magnitude-related activation were also observed in multiplication. Finally, Agostini et al. [19] provided a review on domain-general cognitive skills in children with and without DD. Evaluating 46 studies comparing children with and without DD, the authors reported that primarily executive functions, attention, and processing speed seem to be compromised in DD.

### 4.2. Acquired Acalculia

A last set of studies explored potential atypical numerical processing in adults, for instance, with *acquired acalculia* (AA). In a first study, Danesin and colleagues [20] investigated whether financial decision-making (FDM) was predicted by more basic financial abilities (FA) in neurological patients. Compared with healthy controls, patients with mild cognitive impairment were found to display poorer FDM and FA. By contrast, these abilities were relatively well preserved in patients with Parkinson’s disease and stroke patients. These mixed findings suggest that, all in all, financial decision-making is a complex ability, which is only partially inferable from other financial abilities. Gosling et al. [21] examined the relationship between subitizing and arithmetical abilities in neurological patients with left- or right-hemispheric stroke or posterior cortical atrophy. While no overall correlations were observed, a subitizing impairment was found in two patients who demonstrated very different levels of preserved addition skills. The authors suggest that this dissociation and the large inter-individual variability supports a more componential view of arithmetical ability in AA (thus mimicking the relevant literature focusing on DD). Finally, Berteletti and colleagues [22] examined whether experience with signed language impacts the neurocognitive processes in mental arithmetic using EEG in native signers and hearing speakers. Their findings showed that in native signers and hearing participants alike the neurocognitive processes of subtraction and multiplication are clearly dissociable, but comparable across the two groups.

## 5. Conclusions

As documented by this broad range of studies dealing with different aspects of math learning across the lifespan—from behavioral performance to underlying neural substrates, from cross-sectional to longitudinal evaluations, from healthy to clinical populations—the current Special Issue brought together the expertise of researchers from different backgrounds and clearly contributes to a better understanding of numerical cognition—a topic with both scientific and every-day relevance.
